# Balancing Permeability and Stability: A Study of Hybrid Membranes for Synthetic Cells Using Lipids and PBd-*b*-PEO Block Copolymers

**DOI:** 10.1021/acs.biomac.4c01651

**Published:** 2025-04-08

**Authors:** Caterina Presutti, Edo Vreeker, Sajitha Sasidharan, Zanetta Ferdinando, Marc Stuart, Joanna Juhaniewicz-Dębińska, Giovanni Maglia, Wouter H. Roos, Bert Poolman

**Affiliations:** 1 Department of Biochemistry, University of Groningen, Nijenborgh 3, Groningen 9747 AG, The Netherlands; 2 Chemical Biology, University of Groningen, Nijenborgh 7, Groningen 9747 AG, The Netherlands; 3 Molecular Biophysics, University of Groningen, Nijenborgh 3, Groningen 9747 AG, Netherlands; 4 Electron Microscopy Group, University of Groningen, Nijenborgh 7, Groningen 9747 AG, The Netherlands; 5 Faculty of Chemistry, Biological and Chemical Research Centre, University of Warsaw, Żwirki i Wigury 101, Warsaw 02-089, Poland

## Abstract

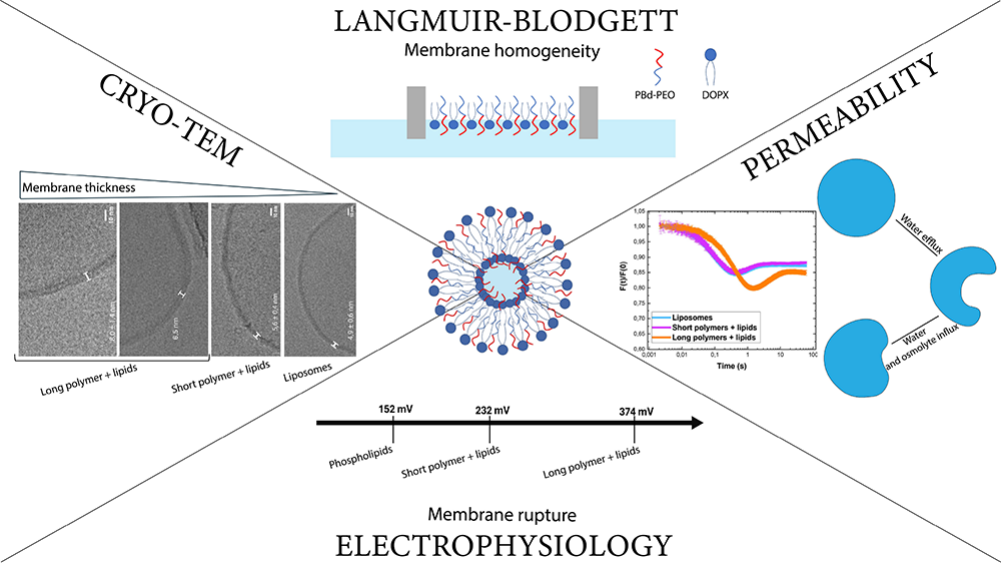

We have synthesized hybrid membranes composed of amphiphilic block copolymers, polybutadiene–poly(ethylene oxide) [PBd-*b*-PEO], with different lengths [PBd_22_-PEO_14_ and PBd_11_-PEO_8_] and mixtures of phospholipids (DOPC:DOPG:DOPE 50:25:25 mol %) to combine the properties of both in terms of stability and fluidity of the membrane. The amphiphilic block copolymers increase the stability, whereas the lipids support the functionality of membrane proteins. The hybrid nature of the bilayers was studied by means of Cryo-TEM, Langmuir–Blodgett technique, atomic force microscopy (AFM), electrical measurements, and fluorescence-based stopped-flow assay to determine the permeability of the membrane for water and osmolytes. We observe that the structural, thermodynamic, and permeability properties of hybrid PBd_11_-PEO_8_ membranes are similar to their purely lipid counterparts, with the advantage of being more stable and resisting a higher transmembrane electrical potential. Hybrid membranes with the longer polymer, PBd_22_-PEO_14_, display more significant structural, thermodynamic, and permeability differences and show less favorable properties than hybrid-PBd_11_-PEO_8_ membranes.

## Introduction

The development of stable membranes as platforms for biosensing, DNA and protein sequencing, or nanocarriers for drug delivery can benefit from the use of membrane lipids with amphiphilic block copolymers. Similarly, the development of synthetic cells with out-of-equilibrium reaction networks requires stable membranes that enable efficient transport of small molecules.^1,2^ Specific lipids are required for membrane (transport) protein function, and amphiphilic block copolymers can stabilize the membrane. Amphiphilic copolymers are molecules based on a hydrophobic and one (AB-type, diblock) or two (ABA-type, triblock) hydrophilic blocks.^3^ The self-assembly of amphiphilic polymers can generate supramolecular assemblies, such as micelles,^4^ tubes,^5^ worm-like structures,^6^ and vesicles.^7−9^

In order to provide a membrane with a biological function, it is essential to be able to reconstitute membrane proteins (transporters, channel proteins, nanopores, lipid synthesizing enzymes, and others) into it. The thickness of the membrane must be similar to that of natural membranes (approximately 4–5 nm) to minimize the hydrophobic mismatch between the amphiphiles and the transmembrane domain of the protein. Furthermore, the membrane must possess a certain fluidity necessary for the conformational dynamics and or translational diffusion of the embedded molecules.^10,11^ In addition, membrane proteins typically have specific lipid (headgroup) requirements.^12,13^ The higher molecular weight of the amphiphilic copolymers and entanglement of the hydrophobic blocks drastically improve the stability of the membranes,^14^ but it leads to thicker and less flexible membranes. Therefore, in order to find a compromise between biocompatibility and fluidity versus stability, lipid–polymer hybrid assemblies may offer benefits from both types of amphiphiles.^15−19^

Several amphiphilic block copolymers have been mixed with lipids, but the most studied in the context with membrane proteins are polybutadiene–poly(ethylene oxide) (PBd-*b*-PEO) block copolymers.^20,21,16,7^ Hybrid membranes are formed when these polymers are mixed with lipids, and they can have properties distinct from those of pure polymersomes and pure liposomes. For example, when mixing PBd_22_–PEO_14_ with POPC to form GUVs, the membrane fluidity decreases with increasing polymer fraction; the diffusion coefficient of the fluorescent lipid probe DiO dropped by ∼55% when the fraction of PBd_22_–PEO_14_ was increased from 0 to 0.25, implying that even a small amount of the polymer can have a high impact on the overall fluidity and possibly other physicochemical properties of the membrane.^22^

PBd_22_-PEO blended with POPC lipids has resulted in the functional membrane reconstitution of cytochrome *bo*_*3*_,^23^ F_O_F_1_-ATP synthase,^7^ ATP-binding cassette (ABC) transporter,^7^ and efflux pump NaAtm1.^24^ Cytochrome *bo*_*3*_ was not functional in PBd_22_-PEO membranes, but ∼80% activity was observed in hybrid membranes with 50% PBd_22_-PEO. Moreover, after 41 days, hybrid vesicles exhibited more than 40% of their original activity, whereas the activity of cytochrome *bo*_*3*_ in lipid vesicles dropped by 97%.^23^ Hybrid membranes could also offer benefits for the bottom-up synthesis of minimal life-like systems.^19,25^

The coassembly of amphiphilic block copolymers with lipids requires tuning not only the fluidity and stability but also the permeability of the membrane. Using a calcein acetomethoxyl-ester based assay, it has been shown that hybrid membranes composed of 50:50 mol % or PBd_22_-PEO:POPC have an intermediate permeability compared to those of pure lipid and pure polymer vesicles. The hybrid EcCL (*E. coli* crude lipid extract and cholesterol)/PBd-PEO (50:50) vesicles had decreased calcein acetomethoxyl-ester permeability in comparison to liposomes.^24^ A number of groups have investigated the proton permeability of hybrid membranes.^7,26,27^ Kleineberg et al.^7^ showed that PBd_22_/POPC hybrid vesicles have a slightly higher permeability than pure lipid vesicles, which aligns with observations of Paxton et al.^26^ The increased permeability has been attributed to a hydrophobic mismatch between lipids and polymers such as PBd_37_-PEO_22_, which may lead to the spontaneous formation of pores or defects in the membrane that facilitate the proton passage.^26^ Similarly, hybrid PBd_22_/POPC membranes with 25% PBd display proton permeability faster than that of pure POPC membranes, whereas membranes with 50% and 75% PBd show reduced permeability. Here, the shorter PBd_22_-PEO_14_ copolymer minimizes hydrophobic mismatch and defects in the membrane.^27^

We now present how polybutadiene–poly(ethylene oxide) (PBd-*b*-PEO) amphiphilic block copolymers with different lengths and molecular weights (PBd_22_-PEO_14_ and PBd_11_-PEO_8_) affect the structure, mechanical and electrical stability, thermodynamic properties, and permeability of vesicles when mixed with phospholipids. We used DOPC:DOPG:DOPE 50:25:25 mol % as a benchmark as this lipid mixture supports the activity of a wide range of membrane (transport) proteins.^1,28,29^

## Materials and Methods

### Materials

Lipids were purchased from Avanti Polar Lipids (Alabaster, AL). The following lipids were used: 1,2-dioleoyl-*sn*-glycero-3-phosphocholine (DOPC), 1,2-dioleoyl-*sn*-glycero-3-phosphoethanolamine (DOPE), and 1,2-dioleoyl-*sn*-glycero-3-phospho-(1′-rac-glycerol) sodium salt (DOPG). Diblock copolymers poly(1,2-butadiene-*block*-ethylene oxide) (PBd-*b*-PEO) of two different molecular weights (PBd_22_–PEO_14_, Mw = 1800 g mol^–1^ and PBd11–PEO8, Mw = 900 g mol^–1^) were purchased from Polymer Source, Inc. (Montreal, Canada). The hypertonic solutions used for the permeability measurements were prepared from potassium chloride (pro analysis; BOOM, Meppel, Netherlands), sodium formate (pro analysis; Merck, Darmstadt, Germany), and glycerol (density 1.26, Ph. Eur., extra pure, BOOM, Meppel, Netherlands).

### Preparation of Liposomes, Hybrid Vesicles, and Polymersomes

Liposomes were prepared using the following synthetic lipid mixture: DOPC:DOPG:DOPE (50:25:25 mol %). Hybrid vesicles were prepared by mixing DOPG:DOPE lipids in a 1:1 molar ratio with either PBd_22_-PEO_14_ or PBd_11_-PEO_8_, while polymersomes were prepared using the diblock copolymers PBd_22_-PEO_14_ and PBd_11_:PEO_8_ at 100 mol %. For the liposome preparation, lipids (25 mg/mL DOPC, DOPG, and DOPE) dissolved in chloroform were mixed at a 50:25:25 molar ratio to a final concentration of 5 mg/mL. For the hybrid vesicle preparation, lipids (25 mg/mL DOPG and DOPE) and diblock copolymers (25 mg/mL) were mixed at a 50:25:25 molar ratio to a final concentration of 5 mg/mL. For the polymersomes, each diblock copolymer, PBd_22_-PEO_14_ (25 mg/mL) and PBd_11_-PEO_8_ (25 mg/mL), was dissolved in chloroform to a final concentration of 5 mg/mL. After the mixtures were prepared, the organic solvent was removed by evaporation with a rotary vaporizer (Rotovapor r-3; BUCHI, Flawil, Switzerland), and the lipid, hybrid, and polymeric film created at the bottom of the flask was finally rehydrated in assay buffer (100 mM KPi pH 7.0) to a concentration of 5 mg/mL, at 40 °C, with gentle rotation for 5 min for the liposomes and 15 min for the hybrid and polymer vesicles. The vesicles were then extruded 13 times through a 200 nm polycarbonate filter.

### Cryo-TEM

Liposomes, hybrid vesicles, and polymersome samples of 2.5 μL (5 mg/mL) were placed on a glow-discharged holey carbon-coated grid (Quantifoil 3.5/1, QUANTIFOIL Micro Tools GmbH). After blotting, the corresponding grid was rapidly frozen in liquid ethane (Vitrobot, FEI) and kept in liquid nitrogen until measurement. The grids were observed with a Gatan model 626 cryostage in a Tecnai T20 FEI cryoelectron microscope operating at 200 keV. Cryo-TEM images were recorded under low-dose conditions on a slow-scan charge-coupled device (CCD) camera.

### Atomic Force Microscopy

#### Sample Preparation

A liquid cell with a glass ring glued to poly-l-lysine-coated coverslips was used for the AFM experiments.^30^ The samples used for the AFM experiments were diluted to a concentration of 0.26 mg/mL. A 20 μL portion of the diluted sample was pipetted onto the poly-l-lysine coverslip. The solution was incubated for 3 min to immobilize the vesicles on the surface, followed by the addition of 580 μL of the imaging buffer (assay buffer, 100 mM KPi pH 7.0).

#### Imaging Conditions

The experiments were performed in liquid at room temperature using a JPK Nanowizard Ultra Speed 1 AFM. The quantitative imaging of the samples was performed using a qp-BioAC CB3 cantilever with a spring constant of 0.03–0.09 N/m. An area of 3 × 3 μm was chosen, and parameters such as an imaging force of 80 pN, a *Z*-length of 400 nm, and a pixel time of 17 ms were maintained throughout the experiments.

#### Data Analysis

The acquired images were postprocessed using the JPK Data processing software version 6.1, following a protocol first described by Vorselen et. al.^31^ A line profile was initially obtained through the maximum data point of the images. As the vesicles deform on the substrate, they are assumed to form a spherical cap. Hence, the height (*H*) and radius of curvature (*R*_c_) of the particles are obtained by fitting a circular arc  in the top half-maximum of the acquired line profile. The term *x* represents the lateral distance data on the *X*-axis, and *x*_0_ is the *X*-coordinate value of the center of the vesicle. The fitting process also deconvolutes the tip radius *R*_t_, for which a value of 10 nm is taken from the data. The radius of the particles in solution (*R*_0_) can be calculated by using the following equation: . The deformation of the particle is calculated by dividing the height data by the radius of curvature. For statistical significance, Welch’s ANOVA test was performed on the deformation data, considering a value of *p* > 0.05.

### Langmuir Monolayer Technique

The surface pressure–area per molecule (π–*A*) isotherms were measured in 100 mM NaPi, pH 7.0, using the Wilhelmy paper plate with an accuracy of 0.1 mN/m. The solutions (15–25 μL) were spread using a micro syringe (Hamilton–Bonaduz, Switzerland) on the buffer subphase, and the solvent was allowed to evaporate over 5 min before starting the compression of the film with the trough barriers moving at a rate of 1.5 cm^2^/min. Each isotherm was repeated at least three times to confirm its reproducibility.

### Liposomes, Hybrid Vesicles, and Polymersomes for Calcein Leakage Experiments

The calcein-filled vesicles were prepared as described previously.^32^ A stock of calcein (from Sigma-Aldrich) was prepared at a concentration of 100 mM in 50 mM KPi, and the pH was adjusted to 7.0 using 5 M NaOH. After mixing the lipids (liposomes), lipids plus polymers in chloroform (hybrid), and polymers (polymersomes) and evaporating the organic solvent with a rotary vaporizer (Rotovapor r-3; BUCHI, Flawil, Switzerland), the lipid, hybrid, and polymer film was rehydrated in 75 mM KPi pH 7.0 plus 10 mM (self-quenching concentration) calcein and mixed by gentle rotation at 40 °C for 5 min (liposomes) and 15 min (hybrid and polymer vesicles). The osmolality of the vesicle lumen was ∼190 mosmol/kg, which equals that of the assay buffer (100 mM KPi pH 7.0). After extrusion through a 200 nm polycarbonate filter, the vesicles were separated from free calcein dye on a 22 cm-long Sephadex-G75 (Sigma-Aldrich) column pre-equilibrated with 100 mM KPi pH 7.0. The collected 1 mL fractions containing the calcein-filled liposomes were identified by eye, using an ultraviolet lamp, and diluted in a total volume of 12 mL of 100 mM KPi at pH 7.0.

### Stopped-Flow Experiments to Determine Solute Permeability

Permeability measurements were conducted using a method of Frallicciardi et al.^32^ Briefly, a stopped-flow apparatus (SX20; Applied Photophysics, Leatherhead, Surrey, UK) operated in single-mixing mode was used to measure the changes in the fluorescence intensity kinetics on application of an osmotic upshift to the vesicles filled with calcein. The hypertonic solutions (100 mM KCl in 100 mM KPi pH 7.0, osmolality: ∼390 mosmol/kg; 100 mM Na Formate in 100 mM KPi 1 pH 7.0, osmolality: ∼350 osmol/kg; and 240 mM glycerol in 100 KPi, pH 7.0, osmolality: ∼460 osmol/kg) and the vesicle solutions were loaded each in one syringe and forced first through the mixer (1:1 mixing ratio and 2 ms dead time) and then into the optical chamber (20 μL volume and 2 mm path length). Since the hypertonic solution and vesicles are mixed in the chamber at a mixing ratio of 1:1, the final concentrations of KCl, Na formate, and glycerol are 50, 50, and 120 mM, respectively; the buffer was 100 mM KPi pH 7.0. Calcein was excited at 495 nm, and the emitted light, collected at 90°, was filtered by a Schott long-pass filter and detected by a photomultiplier tube with 10 μs time resolution. The voltage of the photomultiplier was automatically selected and kept constant during each set of experiments. Three acquisitions were performed for each experimental condition. By osmotically shocking the vesicles, the water efflux causes vesicle shrinkage and increases the calcein concentration, thus decreasing the fluorescence signal. When the hypertonic solution contains permeable osmolytes, these molecules diffuse into the vesicles and (partially) restore the volume, which is seen as an increase in the fluorescence. The decrease in fluorescence on water influx provides information about the permeability coefficient for water, whereas the increase in fluorescence at later times yields the permeability coefficient for the (permeable) osmolyte. A detailed description of the model and analysis of the data are presented elsewhere.^32,33^

### Processing of the Data to Obtain Permeability Coefficients

A detailed protocol for the fitting of the stopped-flow kinetic data is given elsewhere.^32^ Briefly, the raw data are preprocessed in MATLAB (R2023a; MathWorks, Natick, MA). First, the *N* curves, where *N* represents the number of acquisitions (*N* = 3), which we call *f*_*i*_(*t*), acquired in a given experimental condition are averaged (*F*(*t*) = *N*^1–^*∑f*_*i*_(*t*)) and normalized to 1 at time zero (*F*(*t*)/*F*(0)). Second, a fit of the kinetic data is performed to calculate the time evolution of the calcein fluorescence (*F*(*t*)/*F*(0)). The averaged ⟨*F*(*t*)/*F*(0)⟩ curves, along with the vesicle size distribution profile, determined by dynamic light scattering (DLS, see below), are fed to the fitting routine script ‘Ft_ODEfit.m’, which computes the relaxation curves of the calcein concentration by numerical solution of the system of differential equations describing the dynamics of a spherical vesicle on osmotic upshift. Using the Stern–Volmer equation with the dynamic quenching constant *K*_SV_, the numerical solution is used to calculate *F*(*t*)/*F*(0). After feeding the fitting script with the relaxation curves and the vesicle distribution profile, the p*K*_A_ of the acid and the osmolyte concentration must be set to start the fitting routine. For impermeable osmolytes, such as KCl, two fitting parameters are used: *P*_w_ (water permeability coefficient, cm/s) and the quenching constant *K*_SV_ (M^–1^). For permeable osmolytes such as weak acids, Na formate, p*K*_a_ 3.75 (PubChem database, compound ID: 284), and glycerol, p*K*_a_ 14.4 (PubChem database, compound ID: 753), the water permeability coefficient *P*_w_ is fixed to the value obtained with the membrane impermeable osmolyte, whereas *K*_SV_ and *P*_O_ (osmolyte permeability coefficient, cm/s) are obtained from the fitting of the data. We repeat the fitting routine at least 10 times using each of the 10 size distributions acquired by DLS. Data are analyzed using one-way ANOVA followed by Tukey’s multiple-comparisons test.

### Size Distribution of Vesicles

The size distribution of the vesicles was measured by DLS using a DynaPro NanoStar Detector (Wyatt Technology, Santa Barbara, CA). For the DLS measurements, the vesicles were diluted with 100 mM KPi, pH 7 to a concentration range of 2 μg/mL to 2 mg/mL. Measurements were performed with a scattering angle of 90°. For each measurement, at least 10 acquisitions of 20 s each were performed at a temperature of 20 °C. For each acquisition, at least 1.5 million counts were recorded. The correlation curves and intensity-weighted distributions were obtained with the built-in analysis software.

### Electrophysiology Assay

The electrophysiology setup comprises an Axopatch 200B patch clamp amplifier and a DigiData 1440 A/D converter using Clampex 10.7 (Molecular Devices) software to monitor and analyze the experiments. Measurements were performed with 10 kHz sampling frequency and 2 kHz Bessel lowpass filter.

Experiments were carried out using an in-house fabricated device containing two separate chambers (labeled *cis* and *trans*, maximum volume 700 μL each), which are mounted together but separated by a 25 μm-thick Teflon sheet. Free-standing, planar bilayers were formed in an aperture (100–150 μm in diameter) present in the Teflon interface using the Montal–Mueller method.^34,35^ In brief, 20 μL of the lipid/hybrid mix in chloroform was added to each chamber, and the solvent was allowed to evaporate. After evaporation, the Teflon layer was wetted with 5 μL of 4% v/v hexadecane in pentane, and 400 μL of buffer (1 M KCl, 15 mM Tris, pH 7.5) was added to each chamber. Membranes were then formed by subsequently lowering and raising the liquid/air interface past the aperture, resuspending the film of amphiphiles from the bottom of the chamber until a bilayer was formed. The presence of a sealed bilayer was determined with a pair of Ag/AgCl electrodes, with one electrode inserted in each chamber, allowing one to monitor the capacitance of the formed membranes. Formation of a bilayer was confirmed by voltage-induced rupture (1.3 V), followed by rapid reformation of the membrane.

## Results and Discussion

### Morphology and Structure

**Figure 1:**
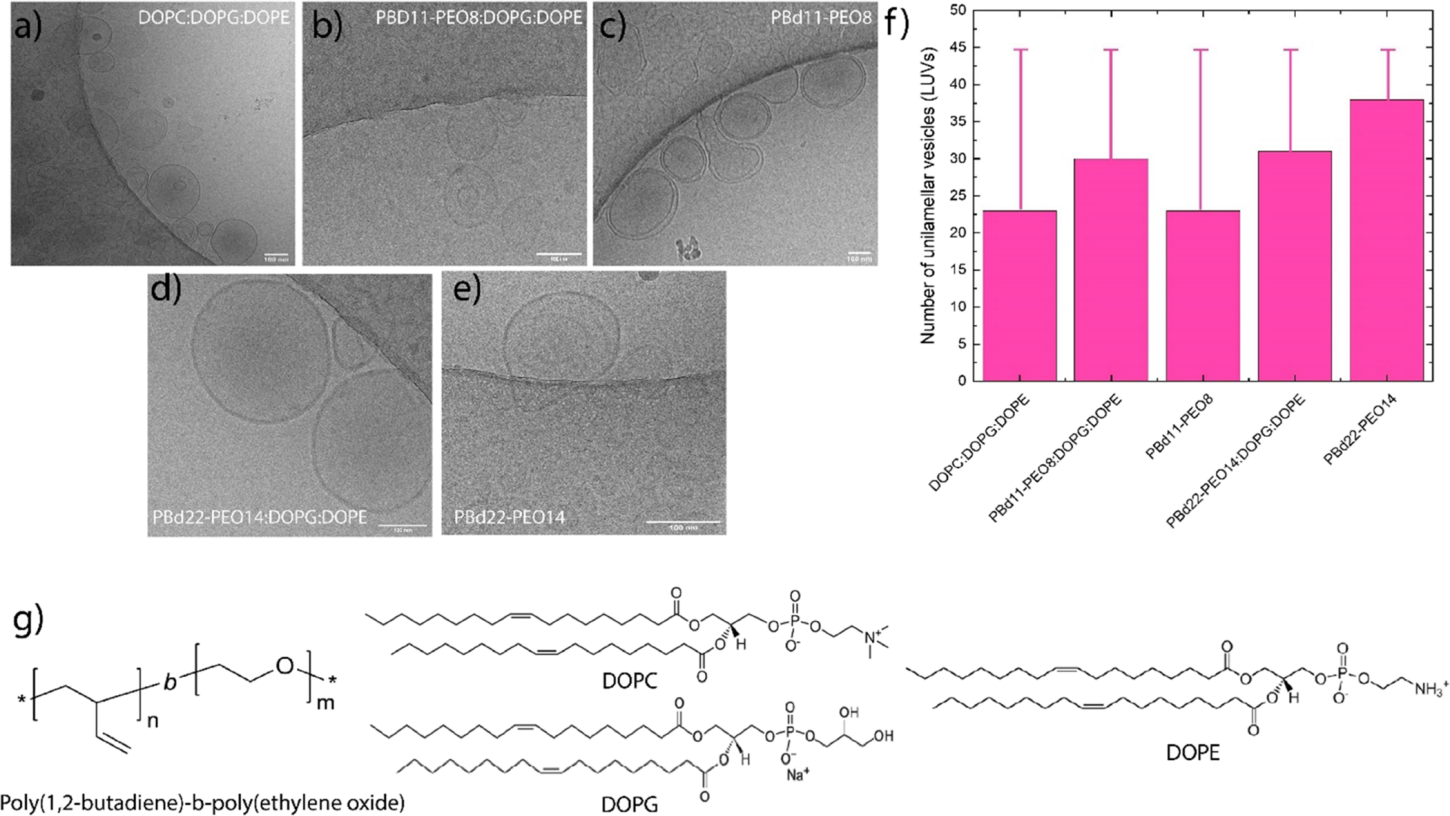
Cryo-TEM images of liposomes, hybrid vesicles, and polymersomes. (a) Liposomes: DOPC:DOPG:DOPE = 50:25:25 mol %. (b) Hybrid vesicles: PBd_11_-PEO_8_:DOPG:DOPG = 50:25:25 mol %. (c) Polymersomes: 100 mol % PBd_11_-PEO_8_. (d) Hybrid vesicles: PBd_22_-PEO_14_:DOPG:DOPE = 50:25:25 mol %. (e) Polymersomes: 100 mol % PBd_22_-PEO_14_. (f) Unilamellarity of the vesicles for the different membrane compositions (*n* = 45 for each composition). (g) Chemical structures of poly(1,2-butadiene)-*b*-poly(ethylene oxide) (PBd-PEO), DOPC, DOPG, and DOPE.

Cryo-TEM analysis of the vesicles in isotonic media shows spherical, elongated, and pleomorphic structures, and as anticipated from the preparation method (limited mechanical energy was used to prepare the vesicles), most vesicles are unilamellar, but a substantial fraction is multilamellar.^36,37^ Most of the lipid vesicles are found inside the holes of the holey carbon grid, but hybrid vesicles and polymersomes show a higher affinity for the carbon grid than pure lipid membranes (Figure 1).

The vesicles were subjected to manual classification for lamellarity (*n* = 45), and they are classified as unilamellar when a single membrane is seen. Phospholipid (DOPC:DOPG:DOPE) and pure polymeric vesicles composed of 100% PBd_11_-PEO_8_ showed about 50% unilamellarity; the percentage of unilamellar vesicles was 66% for PBd_11_-PEO_8:_DOPG:DOPE and 69% for PBd_22_-PEO_14:_DOPG:DOPE. Polymersomes composed of 100% PBd_22_-PEO_14_ showed the highest percentage of unilamellarity (84%) (Figure 1f). While the lamellarity of vesicles as a function composition and pH of the hydration buffer has been investigated,^38^ not much is known about the effect of membrane lipid/amphiphile composition on the lamellarity. Our analysis suggests that thicker (block copolymer-containing) membranes are more unilamellar.

### Bilayer Thickness, Particle Size, and Deformability

**Figure 2:**
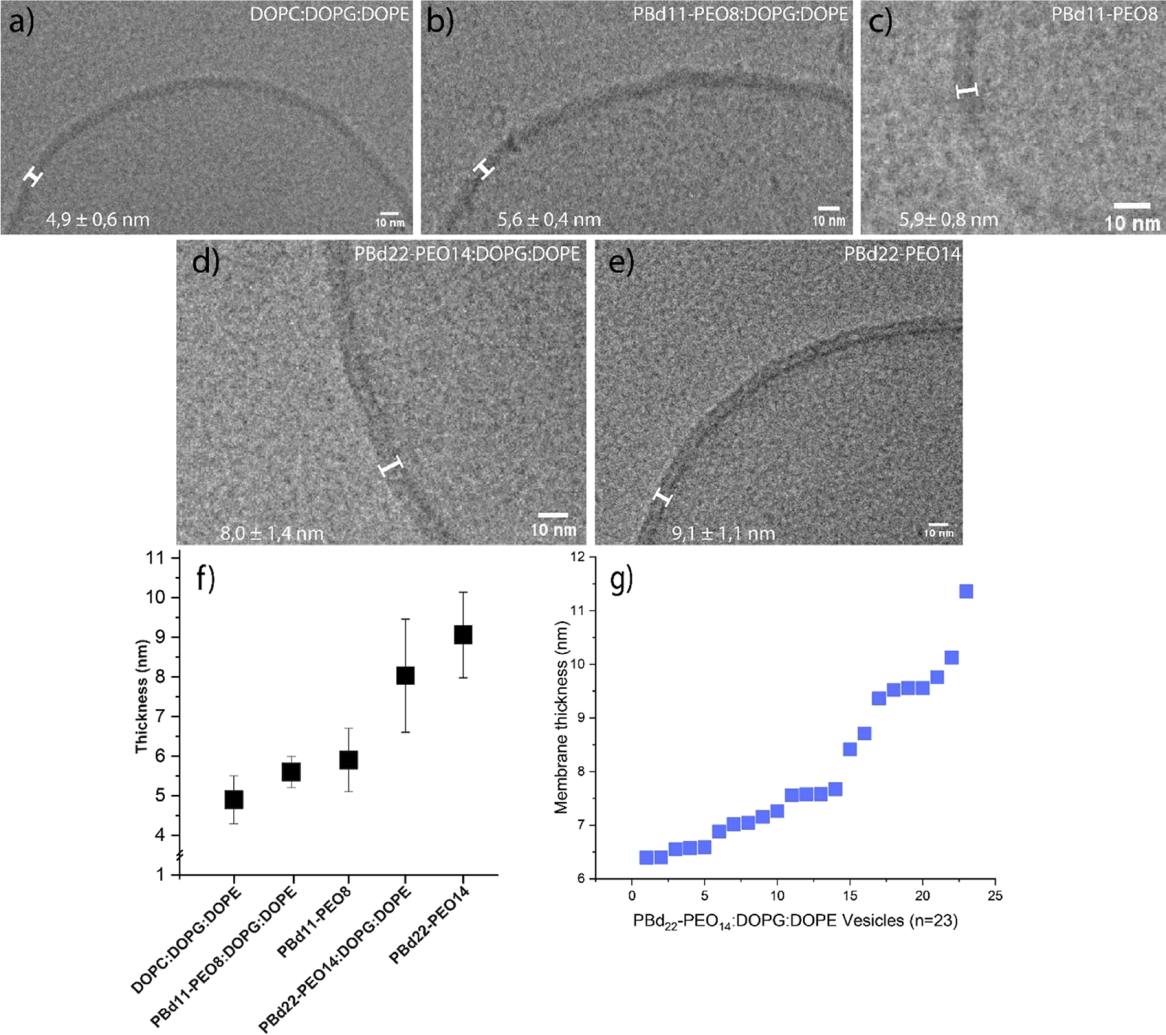
Thickness of the membrane of the vesicles. (a) DOPC:DOPG:DOPE = 50:25:25 mol %. (b) PBd_11_-PEO_8_:DOPG:DOPE = 50:25:25 mol %. (c) 100 mol % PBd_11_-PEO_8_. (d) PBd_22_-PEO_14_:DOPG:DOPE = 50:25:25 mol %. (e) 100 mol % PBd_22_-PEO_14_ 100 mol %. (f) Average thickness of liposomes, hybrid vesicles, and polymersomes. The mean value and the error are the average and standard deviation of *n* = 23 vesicles. (g) Membrane thickness distribution of PBd_22_-PEO_14_:DOPG:DOPE vesicles (*n* = 23).

The thickness of the bilayer, including the hydrophilic and the hydrophobic parts of the amphiphiles, was measured from cryo-EM images for the *n* = 23 vesicles. The thickness of the membrane of DOPC:DOPG:DOPE (50:25:25 mol %) vesicles was 4.9 ± 0.6 nm (Figure 2a, Figure S1), which falls within the range of published values.^39,40^ The hybrid and pure polymer vesicles made of PBd_11_-PEO_8_:DOPC:DOPG and PBd_11_-PEO_8_ had a thickness of 5.6 ± 0.4 and 5.9 ± 0.8 nm, respectively (Figure 2b,c, Figure S1). The thickness is slightly greater than that of the liposomes. The hybrid and polymer vesicles composed of PBd_22_-PEO_14_:DOPG:DOPE and PBd_22_-PEO_14_ have a thickness of 8.0 ± 1.4 and 9.1 ± 1.1 nm, respectively (Figure 2d, f and Figure S1). We note that PBd_11_-PEO_8_ does not affect the thickness of phospholipid vesicles. PBd_22_-PEO_14_, on the other hand, has almost double the thickness, which is in agreement with a recent study where, in the presence of >25 mol % PBd_22_-PEO_14_, the lipids tend to adapt to the thicker hydrophic part of the polymer.^41^ However, differences in the local polymer concentration can also lead to variations along the membrane surface.

**Figure 3:**
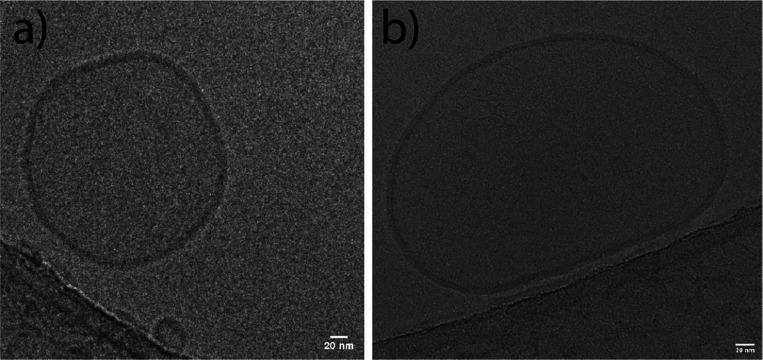
Cryo-TEM images of PBd22-PEO14:DOPG:DOPE = 50:25:25 mol % vesicles displaying two different thicknesses. (a) PBd_22_-PEO_14_:DOPG:DOPE, thickness 9.5 nm. (b) PBd_22_-PEO_14_:DOPG:DOPE, thickness 6.5 nm.

Experimental and computational simulations^41,42^ have shown two distinct vesicle populations for PBd_22_-PEO_14_:POPC (50:50 mol %), one having a thin bilayer, resembling that of liposomes, and another one having a thicker bilayer similar to that of polymersomes. In the thinner membranes, the elastic chains of the polymer tend to adapt to the tighter conformation of the lipids, while in the thicker membrane, the polymer assumes an elongated conformation. Here, the lipid is located at the interface between the hydrophobic and hydrophilic blocks, thereby minimizing the contact with the lipids in the opposite leaflet. This is possible due to differences in local polymer densities; a lower polymer concentration within the membrane leads to a thinner membrane, while a higher local polymer concentration leads to thicker ones.^42^ We found in 5 out of 23 hybrid vesicles, composed of PBd_22_-PEO_14_:DOPG:DOPE, a thickness of around 6.5 nm (Figure 3b) and more than 6.5 nm in 18 out of 23 (Figure 3a). Thus, we also observe differences in thickness, but rather than observing two clear populations, we observe a gradient in the thickness distribution, varying from 6.5 to 11.3 nm (Figure 2g).

The average diameter of the vesicles, as determined by AFM (see Materials and Methods), is between 112.1 and 220.7 nm (Figure S2). We also used AFM to obtain insight into the deformability of the vesicles, which is defined as the ratio of the height (*H*) of the surface-adhered particle and its radius of curvature (*R*_c_). The AFM topographic images of the five samples are shown in Figure S3a–e. The vesicles are spherical in shape, and no particular difference in the morphology was observed across the samples. We find a significant difference in the deformation of liposomes (0.77 *H*/*R*_c_) relative to PBd_22_-PEO_14_ (1.05 *H*/*R*_c_) vesicles, implying that liposomes tend to deform more than the polymeric vesicles. This could either be due to liposomes exhibiting a softer membrane and therefore deforming more as a result of the force applied during AFM imaging or due to an increased adhesion force between liposomes and surface, as compared to polymeric vesicles and the surface. The latter would also yield higher deformability of the liposomes.

To test the adhesive strength of the particles to the surface, imaging at increasing forces was performed. The imaging force was increased in steps of 30 pN.^43^ Due to the increased forces, the particles tend to disrupt from the surface after a while; however, some particles stay attached all the time. We found that 79% of the liposomes detached from the surface, in comparison to only 45% of the PBd_22_-PEO_14_ particles. In other words, the polymer vesicles adhere more strongly to the surface than the liposomes. This shows that the increased deformability of the liposomes is not due to stronger adhesion. Rather, it is most likely due to liposomes displaying a softer membrane than that of the polymer vesicles.

### Langmuir Monolayers

#### Single Amphiphile Monolayers

**Figure 4:**
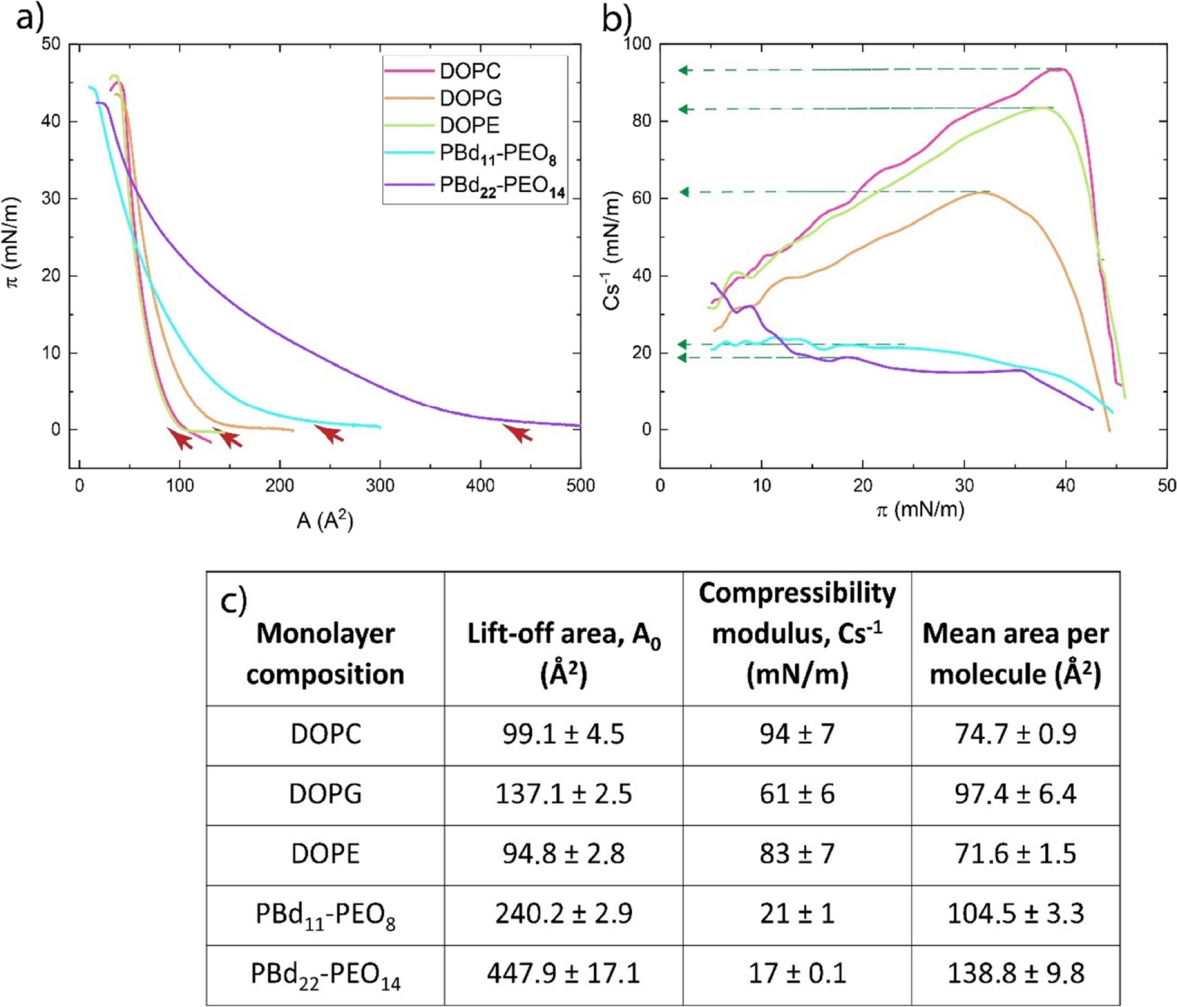
(a) Surface pressure–area isotherms for the single monolayers. (b) Compression modulus–surface pressure graphs for the single monolayers. (c) Characteristic parameters of the monolayers at the air–buffer interface. Data are presented as mean ± standard deviation of *n* = 3 replicates.

Each of the amphiphiles (DOPC, DOPG, DOPE, PBd_11_-PEO_8_, and PBd_22_-PEO_14_) forms Langmuir monolayers at the air–water interface with characteristic isotherms (Figure 4a). The phospholipid isotherms are in agreement with the published data.^44−46^ We analyzed the π/*A* curves obtained for each component with respect to the lift-off area (*A*_0_),^44^ that is, the first value for the area per molecule at which molecules start to interact and the point where surface pressure can be detected (red arrows in Figure 4a). A high *A*_0_ value indicates that the molecules are likely to have weaker intermolecular interactions or bulky structures that prevent tight packing at the interface. For the lipids, *A*_0_, _DOPC_ = 99.1 Å^2^, *A*_0_, _DOPE_ = 94.8 Å^2^, and *A*_0_, _DOPG_ = 137.1 Å^2^. The lift-off area of the amphiphilic block copolymers is higher and increases with an increasing degree of polymerization: *A*_0_, _PBd11-PEO8_ = 240.2 Å^2^; *A*_0_, _PBd22-PEO14_ = 447.9 Å^2^. The high lift-off area of the amphiphilic block polymers indicates that their structures are bulkier and more flexible than those of phospholipids. The lift-off area is shifted toward higher values when the degree of polymerization of the hydrophilic and hydrophobic blocks (PBd_22_-PEO_14_) is increased (Figure 4a), which increases the surface density of PEO blocks. This is in agreement with the mean molecule area values, which we estimate by extrapolating the tangent of the isotherm from the collapse pressure to its relative value of the molecule area (Figure 4c).

To obtain information about the packing and ordering of the molecules in the monolayer, the compression modulus (Cs^–1^) (Figure 4b,c) was calculated using the following equation:
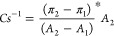
1

The maximum values of Cs^–1^ correspond to the most compressed state of the monolayer that is manifested as the “highest peak” point of the Cs^–1^ vs π graph (green arrows in Figure 4b). A higher compression modulus value corresponds to less compressible membranes.^47^ DOPC, DOPG, and DOPE display the highest Cs^–1^ values (Cs^–1^_DOPC_: 94, Cs^–1^_DOPG_: 61, Cs^–1^_DOPE_: 83), while the two amphiphilic block copolymers display the lowest values (Cs^–1^_PBD11-PEO8_: 21, Cs^–1^_PBD22-PEO14_: 17). In agreement with the lift-off area values, the low compression modulus values of the diblock copolymers indicate that they tend to be more loosely packed and significantly more compressible than the lipid monolayers.

Associated with the packing of the molecules, Cs^–1^ also provides information on the physical state of the monolayer. According to Davies in the 1960s, a Cs^–1^ value in the range of 0–12.5 mN/m corresponds to a monolayer in the gas state (G), 12.5–50 mN/m to the liquid-expanded (LE) state, and 100–250 mN/m to the liquid-condensed (LC) state.^48^ Here, the isotherms and Cs^–1^ values indicate that all the monolayers are in the LE phase, as reported previously for DOPC, DOPG, and DOPE.^44−46^ DOPC, DOPG, and DOPE have the same acyl (oleoyl) chains with one unsaturated bond, so the differences in their physical states are due to their headgroups. Zwitterionic DOPC and DOPE have similar isotherm and compression moduli, while the anionic DOPG shows distinct behavior due to its negative charge, affecting its packing and interactions at the interface. The negatively charged headgroup of PG occupies a larger molecular area (mean molecular area _DOPG_: 97.4 Å^2^) than PE (71.6 Å^2^) and PC (74.7 Å^2^), and electrostatic repulsions lead to a more loose packing of the monolayer than with PC and PE. The compression modulus of the block copolymers is much lower than that of the lipids, which we believe is due to their inherent flexibility and less organized packing. Block copolymers are more amorphous and lack molecular order compared with lipids, which form more structured monolayers and experience phase transitions that raise Cs^–1^ with surface pressure. This enables the polymer chains to constantly reorganize themselves under rising surface pressure without condensation. As a result, the monolayer does not stiffen, and the compression modulus remains low and constant. The absence of a clear phase transition explains why the copolymer’s Cs^–1^–π graph does not show a maximum.

#### Ternary Amphiphile Monolayers

**Figure 5:**
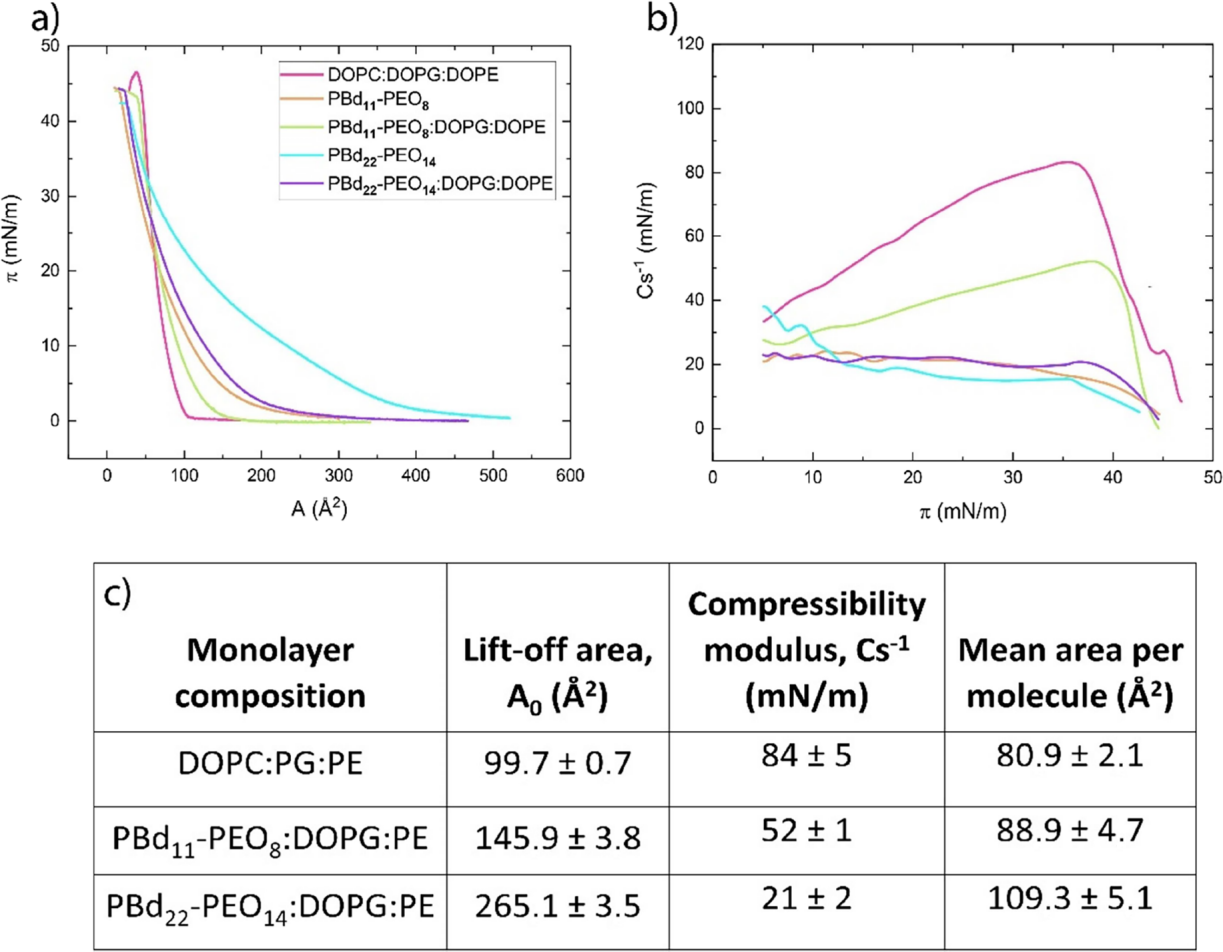
(a) Surface pressure–area isotherms plot. (b) Compression modulus–surface pressure plot of the ternary monolayers (DOPC:DOPG:DOPE, PBd_11_-PEO_8_:DOPG:DOPE, PBd_22_-PEO_14_:DOPG:DOPE) and single monolayers (PBd_11_-PEO_8_ and PBd_22_-PEO_14_). (c) Characteristic parameters of the monolayers at the air–buffer interface. Data are presented as mean ± standard deviation of *n* = 3 replicates.

The π–A isotherms of monolayers composed of mixtures of amphiphiles (DOPC:DOPG:DOPE = 50:25:25 mol %, PBd_11_-PEO_11_:DOPG:DOPE = 50:25:25 mol %, and PBd_22_-PEO_14_:DOPG:DOPE = 50:25:25 mol %) show the LE phase characteristics over the whole range of surface pressures (Figure 5a). The lift-off area of the ternary monolayer depends on the main component of the mixture and displays the same trend as the single component (*A*_0, DOPC:DOPG:DOPE_ = 99.7 Å^2^, *A*_0, PBd11-PEO8:DOPG:DOPE_ = 145.9 Å^2^, *A*_0, PBd22-PEO14:DOPG:DOPE_ = 265.1 Å^2^). The isotherms of the hybrid monolayers lift off at a higher value than those of pure phospholipid mixtures, but they are lower than those of the polymeric amphiphiles alone (*A*_0_, _PBd11-PEO8_ = 240.2 Å^2^, *A*_0_, _PBd22-PEO14_ = 447.9 Å^2^). This suggests that the presence of both lipids and diblock copolymers results in an intermediate packing behavior in the hybrid monolayer, with the lipids contributing to tighter packing than the copolymers alone. This may also be taken as evidence that the different components mix within the monolayer.

To gain more insights into the packing and ordering of molecules at the air–water interface, the compression modulus was determined using eq1. The Cs^–1^ values of the hybrid monolayers are lower than those of the phospholipid mixtures and higher than those of the pure polymeric monolayers (Cs^–1^_PBd11-PEO8:DOPG:DOPE_ = 52 mN/m, Cs^–1^_PBd11-PEO8=_ 21 mN/m, Cs^–1^_PBd22-PEO14:DOPG:DOPE_ = 21 mN/m, Cs^–1^_PBd22-PEO14_= 17 mN/m) (Figure 5b,c. Figure 4c). This suggests that the hybrid monolayers exhibit a more ordered structure than the pure polymeric ones, likely due to the phospholipids that enable tighter packing. However, the Cs^–1^ values of the hybrid monolayers remain lower than those of the phospholipids alone, indicating that the presence of the polymers disrupts full lipid-like ordering, leading to an intermediate state of compressibility. This intermediate compressibility reflects the balance between the flexibility of the polymers and the ordering effects of the phospholipids. Moreover, the decrease in Cs^–1^ indicates that the block copolymers increase the disorder and lower the packing of the monolayer.

For the PBd_11_-PEO_8_:DOPG:DOPE hybrid, there is an appreciable difference in the compressibility moduli of the pure phospholipid and pure polymer monolayers (Cs^–1^_DOPC:DOPG:DOPE_ = 84 mN/m, Cs^–1^_PBd11-PEO8:DOPG:DOPE_ = 52 mN/m, Cs^–1^_PBd11-PEO8_ = 21 mN/m), while the compressibility moduli of the PBd_22_-PEO_14_:DOPG:DOPE hybrid and PBd_22_-PEO_14_ are similar (Figure 4c, Figure 5b,c) (Cs^–1^_PBd22-PEO14:DOPG:DOPE_ = 21 mN/m, Cs^–1^_PBd22-PEO14_ = 17 mN/m). An intermediate Cs^–1^ value, as observed for the hybrid PBd_11_-PEO_8_:DOPG:DOPE, implies that the system is more disordered than the pure lipid mixture but more packed than the pure polymer mixture and most likely well mixed. We also note that PBd_22_-PEO_14_ has a larger mass than PBd_11_-PEO_8_, and at an equal molar ratio, the larger polymer may dominate the physical properties of the hybrid membranes more than the smaller one, reducing the contributions from the lipids. In fact, the properties of the hybrid membranes with PBd_22_-PEO_14_ mimic those of the pure polymersomes in terms of mixing (compressibility) and the ultrastructure of the vesicles. The properties of the PBd_22_-PEO_14_:DOPG:DOPE hybrid agree with our cryo-TEM data and previous data of the Jeuken group,^41^ which suggests two populations of vesicles with different thicknesses. In the thinner hybrid membranes, the elastic chains of the polymer tend to adapt to the lipid’s tighter conformation. In contrast, in the thicker membrane, the polymer assumes an elongated conformation, where the contacts between lipids in the opposite leaflet are minimized. Overall, the longest polymer, PBd_22_-PEO_14_, affects the properties of the hybrid monolayer much more than the shorter PBd_11_-PEO_8_ does. We thus think that PBd_11_-PEO_8_ is preferred in future studies with membrane proteins embedded in the hybrid membranes.

#### Miscibility

The ternary mixtures might have different interfacial properties compared to single component monolayers. To gain more insights into the interactions between the different amphiphile components, the excess area of mixing was calculated according to the additivity rule using the following equation:
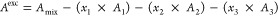
2where *A*_mix_ is the mean molecular area of the mixture at a given surface pressure, *A_n_* is the mean molecular area of one component, and *x_n_* is the molar fraction of the relative component.

The excess area of mixing provides information about the nonideal behavior of a monolayer compared to an ideal mixture. When *A*^exc^ = 0, the components of the monolayer are ideally miscible or totally immiscible, while when *A*^exc^ ≠ 0, the components are partially miscible.^46^ Positive values of *A*^exc^ indicate unfavorable interactions between molecules, where repulsive forces between the components result in looser packing, causing the molecules to occupy more space than predicted for an ideal mixture. This may indicate poor mixing and the potential for phase separation. In contrast, negative *A*^exc^ values indicate that the components mix well with molecules packing more tightly together than expected in an ideal system. This usually implies favorable interactions between the components, such as strong attractive forces or good molecular arrangement. The type of interaction is influenced by two types of effects: attractive forces between the hydrophobic regions of the molecules and positive or negative interactions between the heads of the molecules, which depend on their charge.^49^

The magnitude of these interactions can be obtained from the π/*A* isotherms, which estimates the excess of free Gibbs energy,^49^ Δ*G*^exc^:
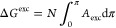
3where *N* is Avogadro’s number.

A negative Δ*G*^exc^ indicates that mixing is thermodynamically favorable with strong attractive interactions between the molecules. A positive Δ*G*^exc^ value indicates that mixing is thermodynamically unfavorable, implying that the components tend to repel each other or prefer to phase-separate. This can lead to instability or heterogeneity in the monolayer.

**Figure 6:**
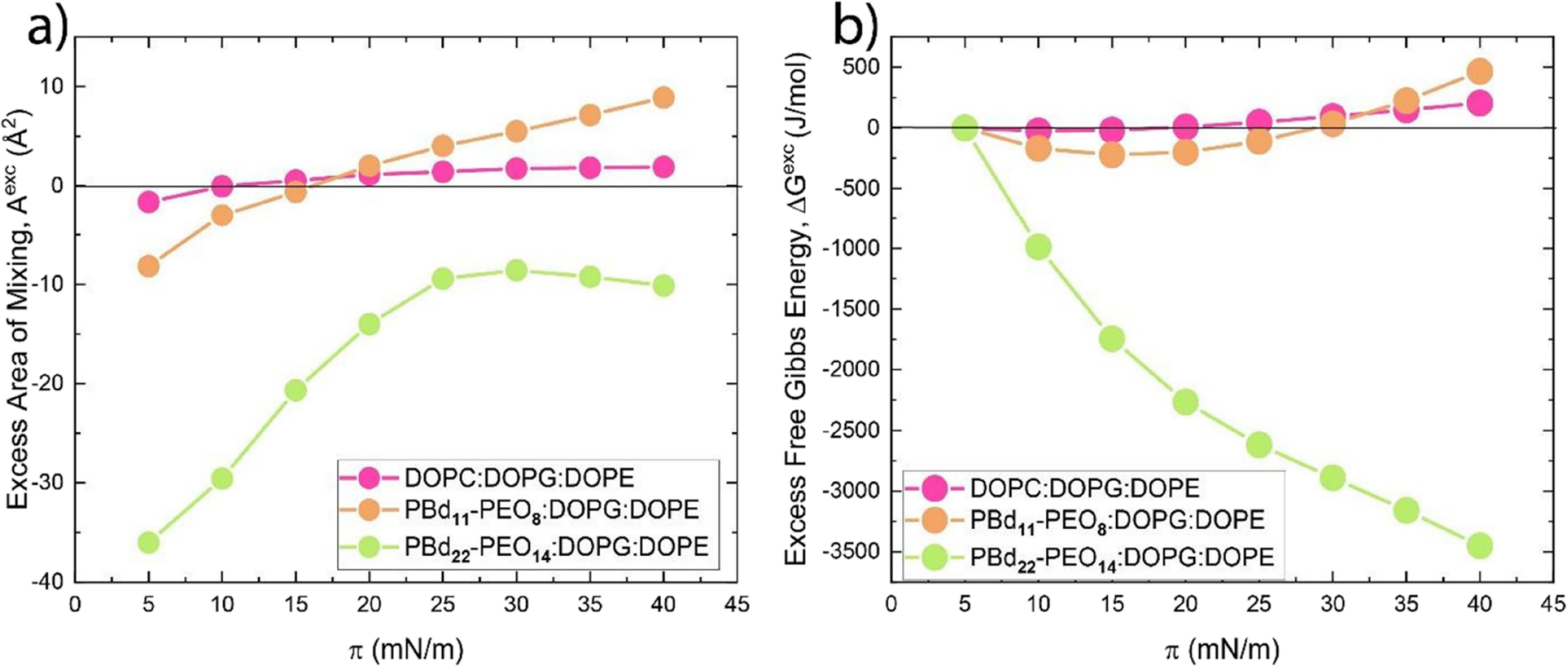
Excess area of mixing–surface pressure (a) and excess free Gibbs energy plots for the ternary monolayers.

As observed for the isotherms, the DOPC:DOPG:DOPE and PBd_11_-PEO8:DOPG:DOPE mixtures display a similar behavior (Figure 6a). Negative values of *A*^exc^ are observed for surface pressures from 5 to 15 mN/m, but then, at higher surface pressures, *A*^exc^ becomes slightly positive for both mixtures. These relatively small deviations suggest that while there is some degree of favorable mixing at lower surface pressures and a slight tendency toward less efficient packing or repulsion at higher pressures, the overall interactions between the components remain balanced without significant phase separation or drastic changes in packing behavior. At 30 mN/m, the values are +1.8 and +5.5 Å^2^ for DOPC:DOPG:DOPE and PBd_11_-PEO8:DOPG:DOPE, respectively. The excess free energy of mixing remains negative up to 15 mN/m for the phospholipid mixture and up to 25 mN/m for the hybrid PBd_11_-PEO_11_:DOPG:DOPE mixture (Δ*G*^exc^_DOPC:DOPG:DOPE at 15 mN/m_ = −17.4 J/mol, Δ*G*^exc^_PBd11-PEO8:DOPG:DOPE at 25 mN/m_ = −109.7 J/mol) (Figure 6b). Overall, we find a slight deviation from ideality for both ternary mixtures, but the values are very low.

For PBd_22_-PEO_14_:DOPG:DOPE, the negative values of *A*^exc^ and Δ*G*^exc^ are observed between 5 and 40 mN/m (Figure 6a,b). The changes in the Δ*G*^exc^ of the hybrid PBd_11_-PEO_8_ mixtures indicate that the stability of the mixed monolayer slightly decreases on compression, while for the hybrid PBd_22_-PEO_14_, the decrease in Δ*G*^exc^ suggests that the stability of the system increases on compression_._

Taking ∼30 mN/m as a surface pressure typical for lipid bilayers,^50,51^ we obtain a slightly positive deviation from ideality (*A*^exc^_DOPC:DOPG:DOPE at 30 mN/m_ = 1.8 Å^2^, *A*^exc^_PBd11-PEO8:DOPG:DOPE at 30 mN/m_ = 5.5 Å^2^) for the phospholipid and hybrid PBd_11_-PEO_8_:DOPG:DOPE mixture, while it is negative (*A*^exc^_PBd22-PEO14:DOPG:DOPE at 30 mN/m_ = −8.6 Å^2^) for the hybrid PBd_22_-PEO_14_:DOPG:DOPE mixture. Therefore, the strongest interactions between amphiphiles are found in the hybrid PBd_22_-PEO_14_ mixtures, indicating that these are the most thermodynamically stable systems. The slightly positive deviation observed at 30 mN/m for the phospholipid mixture and hybrid mixture with PBd_11_-PEO_8_ does not imply phase separation but may indicate a less stable system.

Studies on the phase behavior of PBd-PEO plus unsaturated lipids have shown that they form a homogeneous membrane;^22^ phase separation and poor miscibility occur when PBd-PEO is mixed with saturated lipids such as DPPC.^52^ As noted before, the hydrophobic interactions between the acyl chains and the polybutadiene blocks and attraction or repulsion between the head groups and the poly(ethylene oxide) block are taken into account when *A*^exc^ is determined. With the longer hydrophobic block of PBd_22_-PEO_14_, hydrophobic attractive forces are prevalent, which stabilize the system more. The larger PBd_22_-PEO_14_ molecule may help reduce electrostatic repulsions between charged lipids such as DOPG by increasing the spatial separation between the headgroups and providing some degree of shielding. This spatial separation restricts electrostatic repulsion, further contributing to the overall stabilization of the hybrid monolayer despite the absence of a tightly packed structure. Since the extent of the hydrophobic interactions depends on the acyl chain length,^46^ we attribute the differences in thermodynamic behavior of the two hybrid systems to the extent of the hydrophobic interactions. Moreover, PBd_22_-PEO_14_, being a larger molecule, could reduce the electrostatic repulsions between charged lipids and thereby stabilize the system.

### Electrical Characterization of Planar, Free-standing Bilayers

Next, the electrical properties of the lipid and hybrid membranes were assessed. For this purpose, free-standing, planar bilayers were formed across an aperture (100–150 μm in diameter) in a hydrophobic Teflon substrate according to the Montal–Mueller method (see Materials and Methods for details of membrane formation).^34,35^ The design of the chamber allows the insertion of an Ag/Agcl electrode on each side of the membrane, enabling the application of a transmembrane potential and the monitoring of the electrical properties of the formed membranes.

The capacitance of an amphiphilic membrane is related to its thickness as follows:
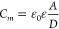
with vacuum permittivity ε_0_ = 8.85410^–12^ F/m, dielectric constant ε, membrane area *A*, and membrane thickness *D*.

Successful formation of a bilayer made of DOPC:DOPE:DOPG (50:25:25 mol %) induced a capacitance value of 160 ± 28 pF (average ± standard deviation, *N* = 5). Substituting DOPC with PBD_11_-PEO_8_ yielded hybrid membranes with capacitance values of 123 ± 9 pF, while with PBD_22_PEO_14_, the membrane capacitance was 106 ± 10 pF (*N* = 5 for both hybrid membranes). Assuming that free-standing bilayers formed across the aperture yield similar values for *A*, the lower capacitance values for PBD_22_-PEO_14_:DOPE:DOPG bilayers relative to PBD_11_-PEO_8_:DOPE:DOPG are indicative of an increase in membrane thickness, which aligns with our cryo-TEM findings.

We then investigated the stability of the bilayer membranes toward applied electrical potentials. Our approach was to assess the reaction of each membrane type toward a voltage ramp, applying a constant transmembrane potential for at least 1 min before increasing the potential with increments of 10 mV (*N* = 10 for each membrane set). Membranes formed with DOPC:DOPE:DOPG showed the lowest stability toward applied potentials, demonstrating electroporation (yielding erratic transient current signals) at 118 ± 27 mV, followed by membrane rupture at 152 ± 23 mV. Hybrid membranes formed with PBD_11_-PEO_8_ polymers underwent electroporation at a slightly higher potential, 132 ± 28 mV, while membranes did not rupture until potentials of 232 ± 64 mV were reached. Substituting DOPC with PBD_22_-PEO_14_ yielded hybrid membranes with greatly improved stability toward the applied potentials, with no electroporation observed and only rupture at 374 ± 19 mV.

For reference, in our earlier work,^53^ we showed that pure PBD_22_-PEO_14_ membranes obtained the highest rupture potential (540 ± 50 mV), while PBD_11_-PEO_8_ polymer membranes ruptured at 420 ± 60 mV, and lipid membranes formed with DPhPC collapsed at 240 ± 40 mV, with no observed electroporation. Additionally, hybrid membranes comprising PBD_22_-PEO_14_ and DPhPC at roughly equimolar concentrations were found to rupture at 540 ± 110 mV, while membranes formed with PBD_11_PEO_8_ and DPhPC had a rupture potential of 420 ± 100 mV. The lower stability of the hybrid membranes studied in this work toward applied potentials may be attributed to the presence of negatively charged lipids (DOPG) and the unsaturated dioleoyl chains.^54^ A destabilizing effect of negatively charged amphiphiles in hybrid membranes has been reported by Koner et al.,^55^ who found that hybrid membranes formed with PBD–PEO–COOH polymers (negatively charged at neutral pH) and DPhPC became leaky and unstable when polymer concentrations exceeding 15 mol % were used.^55^ The enhanced electrical stability of membranes formed with PBD_11_-PEO_8_ and DPhPC compared to PBD_11_-PEO_8_:DOPG:DOPE membranes shows that the polymers only partly compensate for the destabilizing effects of DOPG and DOPE. Hybrid membranes formed with PBD_22_-PEO_14_ have the highest electrical stability, which aligns with our observation that hybrid membranes containing this polymer have a highly reduced change in Gibbs free energy of bilayer formation.

### Permeability of Membranes

#### Physiochemical Description of the Model

The permeability measurements are based on the model described by Gabba and Poolman.^33^ The permeability coefficients of membranes (in cm/s) are obtained by solving a system of equations that describe the relaxation dynamics of the vesicles on osmotic upshift with an impermeable osmolyte such as KCl and permeable osmolytes such as weak acids (Na formate) or alcohols (glycerol). Briefly, the model is based on the following: (1) the membrane is deformable and the vesicles shrink on osmotic upshift; (2) the membrane thickness is much smaller than the vesicle radius; (3) the weak acid strength (p*K*_a_ value) determines the dynamic behavior of the system (only the acid is membrane permeable); and (4) calcein self-quenching follows the volume of the vesicles.^32,33,56^

**Figure 7:**
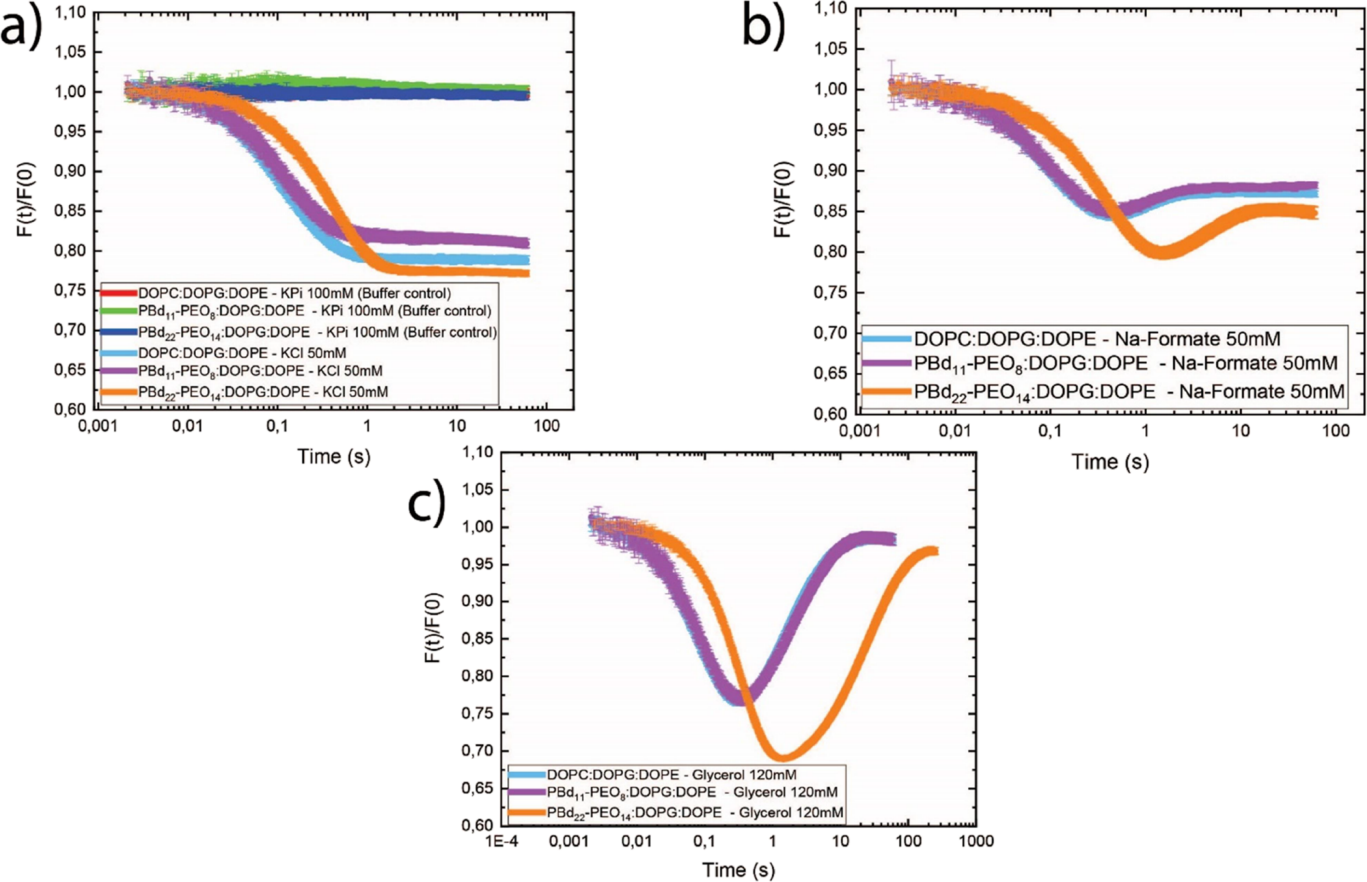
Kinetic data obtained with the calcein self-quenching assay using liposomes (DOPC:DOPG:DOPE) and hybrid vesicles (PBd_11_-PEO_8_:DOPG:DOPE and PBd_22_-PEO_14_:DOPG:DOPE, 50:25:25 mol %) treated with 100 mM KPi pH 7 (buffer control, curve red, green and blue) or upshifted with 50 mM KCl (curve light blue, purple and orange) (a), 50 mM formic acid (b), or 120 mM glycerol (c).

We used vesicles with calcein at a self-quenching concentration of 10 mM,^56^ and the internal and external solutions are equiosmolal (see Materials and Methods). We then osmotically shock the vesicles with a hypertonic solution containing an impermeable osmolyte, which causes shrinkage due to the water efflux and quenching of the calcein fluorescence (Figure 7a). The relaxation dynamic lasts until the internal and external osmolality have become equal, and from the kinetics, we obtain the permeability coefficient for water.^33^ We observe a decrease in fluorescence followed by an increase when a permeable osmolyte such as formic acid (p*K*_a_ = 3.75) or glycerol is used (Figure 7b,c). The recovery of the fluorescence reflects the influx of formic acid (Figure 7b) and glycerol (Figure 7c), and from the kinetics and *P*_H2O_, the permeability coefficients of the osmolytes are obtained. The recovery is only partial with Na formate because Na^+^ ions are impermeable on the time scale of the measurements.

#### Permeability of the Vesicles

**Figure 8:**
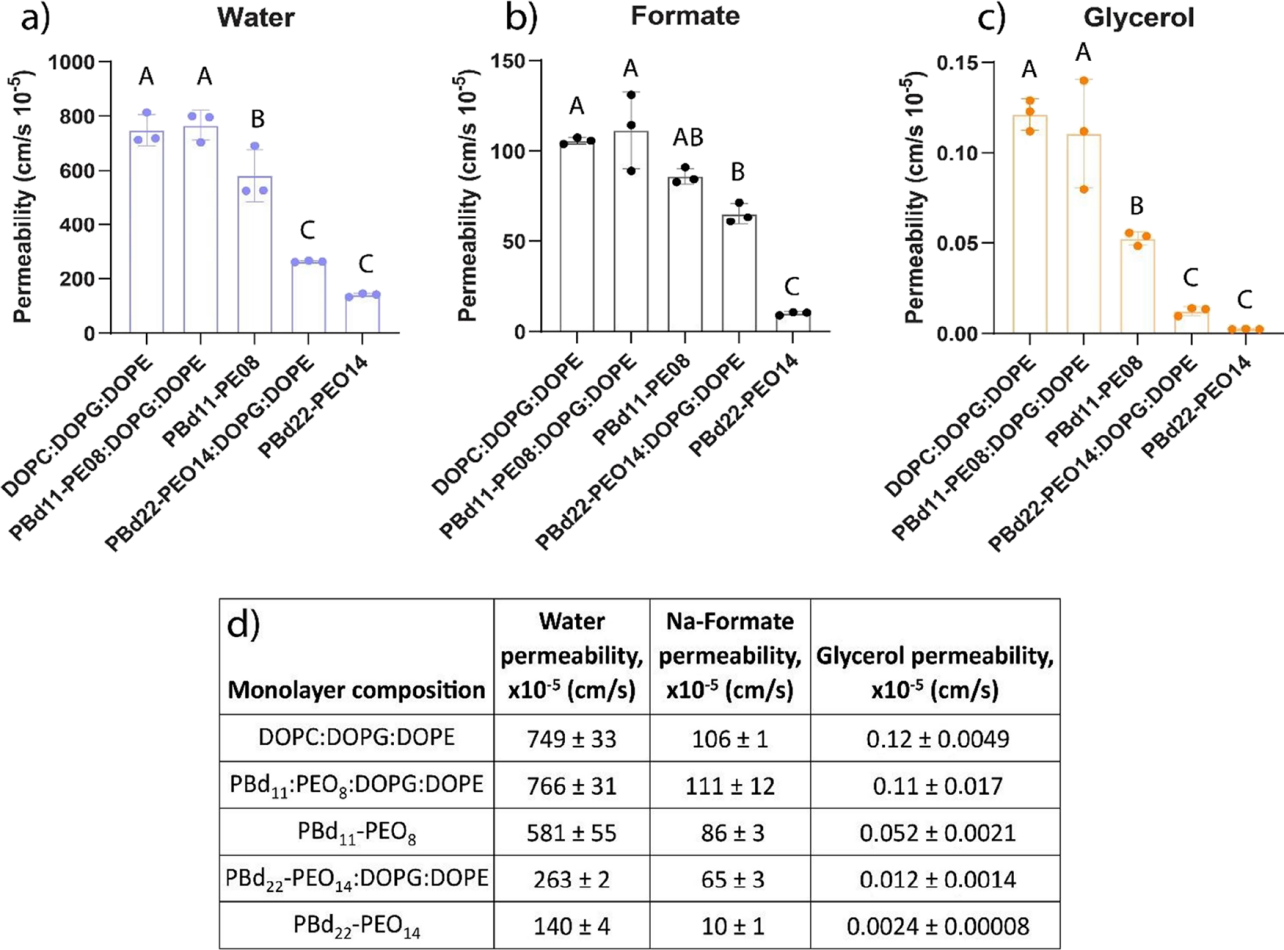
Water (a), formic acid (b), and glycerol (c) permeability coefficients (cm/s) for phospholipid (DOPC:DOPG:DOPE), hybrid (PBd_11_-PEO_8_:DOPG:DOPE and PBd_22_-PEO_14_:DOPG:DOPE), and polymer (PBd_11_-PEO_8_ and PBd_22_-PEO_14_) vesicles. Each point is the average of *n* = 10 permeability coefficients calculated using 10 DLS acquisitions. Three biological replicates were conducted for each membrane composition. Permeability coefficients (cm/s) for water, formic acid, and glycerol in liposomes, hybrid vesicles, and polymersomes (d). Data are presented as mean (*n* = 30) ± s.e.m. Different letters mean significant difference (*P* < 0.05). Data were analyzed using one-way ANOVA followed by Tukey’s multiple-comparisons test.

We do not find a significant difference in the permeability for water, formic acid, and glycerol between phospholipid (DOPC:DOPG:DOPE 50:25:25 mol %) and hybrid PB_11_-PEO_8_:DOPG:DOPE vesicles (Figure 8). However, hybrid PBd_22_-PEO_14_:DOPG:DOPE vesicles have an approximately 3-fold lower permeability for water (Figure 8a), 1.6-fold lower permeability for formic acid (Figure 8b), and 10-fold lower permeability for glycerol (Figure 8c). Thus, the longer hydrophobic block of PBd_22_-PEO_14_ creates a larger permeability barrier, but the effect is significantly different for water, formic acid, and glycerol. The full data sets are shown in Figures S4–S6.

The permeability of the pure polymer is always lower than that of the hybrid membranes (Figure 8). The differences in permeability for water and glycerol between hybrid PBd_22_-PEO_14_:DOPG:DOPE vesicles and PBd_22_-PEO_14_ polymersomes are not statistically significant (Figure 8a,c, indicated by the letter C). Conversely, differences in permeability for formic acid are significant (indicated by B and C in Figure 8b). Of all the vesicles, polymersomes made of PBd_22_-PEO_14_ had the lowest permeability.

We extract some general points from these data: (1) permeability measurements confirm the relatively high solute barrier of (hybrid) membranes, which is crucial for functional studies with, e.g., membrane proteins and the engineering of synthetic cells; (2) PB_11_-PEO_8_ polymer replacing DOPC does not significantly affect the permeability; (3) PBd_22_-PEO_14_ polymer replacing DOPC lowers the permeability; and (4) membranes composed of amphiphilic block copolymers are less permeable for solutes than phospholipid vesicles.

The low permeability seen in pure polymer membranes aligns with previous studies that reported extremely low proton permeability in PBd_54_-PEO_44_ membranes. This study demonstrated that PBd-based membranes can maintain pH gradients of 10 for at least 8 days.^57^ The decrease in permeability with block copolymers in lipid membranes aligns with the findings of Battaglia et al.,^58^ who showed that the permeability of membranes composed of poly(ethylene oxide)-*co*-polybutylene oxide (EB) copolymers correlates with the thickness of the membranes, as predicted by Fick’s first law.^58^ In our experiments, we also observe that increasing the thickness results in a decrease in the permeability. Although a gradient in thickness was observed for the PBd_22_-PEO_14_:DOPG:DOPE, the permeability measurements, which are taken from ensembles of vesicles, average out the local polymer variations seen in cryo-TEM in the stopped-flow kinetic data.

To conclude, while previous studies showed that hybrid membranes made of PBd_22_-PEO_14_/POPC display higher proton permeability than pure lipid membranes,^7,26,27^ here, we show that the presence of block copolymers decreases the permeability of larger molecules such as water, formic acid, and glycerol.

## Conclusions

Hybrid vesicles represent an emerging material in soft matter research due to the potential that they offer in biomedical applications^59,60^ and in synthetic biology,^25^ acting as a platform for the functional reconstitution of membrane proteins.^7,19,23,24^ We now show how polybutadiene–poly(ethylene oxide) (PBd-*b*-PEO) amphiphilic block copolymers with different lengths and molecular weights (PBd_22_-PEO_14_ and PBd_11_-PEO_8_) affect the structure of submicron-size vesicles, their mechanical stability and thermodynamic properties, and the permeability of the bilayer for water and low-molecular-weight osmolytes.^28,29^ The pure phospholipid (DOPC:DOPG:DOPE) membranes, known to enable the activity of complex membrane proteins,^29^ were used as reference to compare the physical and structural properties of hybrid membranes made of PBd_22_-PEO_14_ and PBd_11_-PEO_8_ mixed with DOPG and DOPE.

The cryo-TEM data show that both the pure polymer and the hybrid membranes form spherical vesicles with varying degrees of unilamellarity. While the thickness of the double layer of the phospholipid, hybrid-PBd_11_-PEO_8,_ and pure PBd_11_-PEO_8_ composition falls within the range of 4–6 nm, the presence of PBd_22_-PEO_14_ in the membrane can double the thickness. Moreover, for the hybrid- PBd_22_-PEO_14_ vesicles, the thickness ranges from 6 to 9.5 nm, implying that, in agreement with previous studies,^41,42^ differences in the local polymer concentration affect the local thickness. Despite the heterogeneity, most vesicles had a thickness similar to that of pure polymersomes. In both hybrid assemblies, the homogeneous mixing and the hybrid nature of the membrane have been confirmed by their compressibility modulus (Cs^1–^), whose value falls halfway between that of pure phospholipids and pure polymers. The compressibility modulus provides insights into the packing and ordering of the molecules, and our data show that the molecules tend to be more disordered and less packed with increasing polymer content.

In hybrid membranes, lipids can localize in the interface between the hydrophobic and the hydrophilic block of the polymers rather than forming a more packed conformation where the polymer adapts its structure to the lipid. This would align with our cryo-TEM data, where most of the hybrid-PBd_22_-PEO_14_ vesicles have a thicker bilayer, comparable to that of the corresponding polymersomes. The excess area of mixing and the excess of free Gibbs energy of the phospholipid monolayer are similar for hybrid-PBd_11_-PEO_8_, showing good miscibility between the components of the monolayer. Conversely, as highlighted by the strong negative deviation from ideality, the strongest interactions between lipids and polymers are found in the hybrid-PBd_22_-PEO_14_ vesicles. Since the extent of the attractive interactions mainly depends on the presence of hydrophobic interactions, we attribute the differences in the thermodynamic behavior of the two hybrid systems to the length of the hydrophobic block that stabilizes the system.

Overall, while the presence of PBd_11_-PEO_8_ mixed with lipids does not affect the physical properties of the membrane, the PBd_22_-PEO_14_ block copolymer influences the interactions between the components leading to thicker and more disordered membranes. The greater stability of hybrid-PBd_22_-PEO_14_ membranes compared to the lipid vesicles is shown by their ability to resist rupture under the application of an increasing electrical potential. Replacing PBd_22_-PEO_14_ with DOPC in the hybrid membranes drastically increased the stability of the bilayer toward a potential of up to 374 mV (compared to 152 mV for lipid membranes). Pure polymeric membranes proved to be most stable, as also confirmed by AFM, but they may be less useful for the functioning of complex membrane proteins, requiring specific lipids. Permeability measurements show that PBd_11_-PEO_8_, the shortest block copolymer, does not change the permeation of water, formic acid, and glycerol, while the incorporation of PBd_22_-PEO_14_ lowers the permeability. Overall, we show that the hybrid-PBd_11_-PEO_8_ vesicles have structural and physical properties similar to those of pure phospholipid membranes, with the advantage of increased stability. We propose exploring PBd_11_-PEO_8_ in future studies of hybrid membranes with integral membrane proteins embedded in the bilayer.

## Supporting Information Available

Measurements of bilayer thicknesses in vesicles using cryo-TEM; determination of average vesicle diameters with AFM; deformability tests of vesicles with AFM; permeability assays of vesicles for water, formic acid, and glycerol (PDF)
