# Supplementary material for: Balancing Permeability and Stability: A Study of Hybrid Membranes for Synthetic Cells Using Lipids and PBd-b-PEO Block Copolymers
Source: Biomacromolecules. 2025 Apr 8;26(5):2868–81. doi: 10.1021/acs.biomac.4c01651 (PMC12076510; doi:10.1021/acs.biomac.4c01651)
Supplement: Supplementary file 1 — bm4c01651_si_001.pdf [file bm4c01651_si_001.pdf]

Supplementary Information

Balancing Permeability and Stability: a study of hybrid membranes for synthetic cells using lipids and PBd-b-PEO Block Copolymers

Caterina Presutti(1), Edo Vreeker(2), Sajitha Sasidharan(3), Zanetta Ferdinando(3), Marc Stuart(4), Joanna Juhaniewicz-Dębińska(5), Giovanni Maglia(2), Wouter H. Roos(3), and Bert Poolman(1)

(1) Department of Biochemistry, University of Groningen, Nijenborgh 4, 9747 AG, Groningen, the Netherlands

(2) Chemical Biology, University of Groningen, Nijenborgh 7, 9747 AG, the Netherlands

(3) Molecular Biophysics, University of Groningen, Nijenborgh 4, 9747 AG Groningen, Netherlands

(4) Electron Microscopy group, University of Groningen, Nijenborgh 7, 9747 AG Groningen, The Netherlands

(5) Faculty of Chemistry, Biological and Chemical Research Centre, University of Warsaw, Żwirki i Wigury 101, 02-089 Warsaw, Poland

Membrane composition	Molar ratio (mol%)	Thickness (nm)
DOPC:DOPG:DOPE	50:25:25	4,9 ± 0,6
PBd11-PEO8:DOPG:DOPE	50:25:25	5,6 ± 0,4
PBd11-PEO8	100	5,9 ± 0,8
PBd22-PEO14:DOPG:DOPE	50:25:25	8,0 ± 1,4
PBd22-PEO14	100	9,1 ± 1,1

Figure S1. Thickness of the bilayer of liposomes, hybrid vesicles, and polymersomes. The mean value and the error are the average and standard deviation of n=23 vesicles

a)

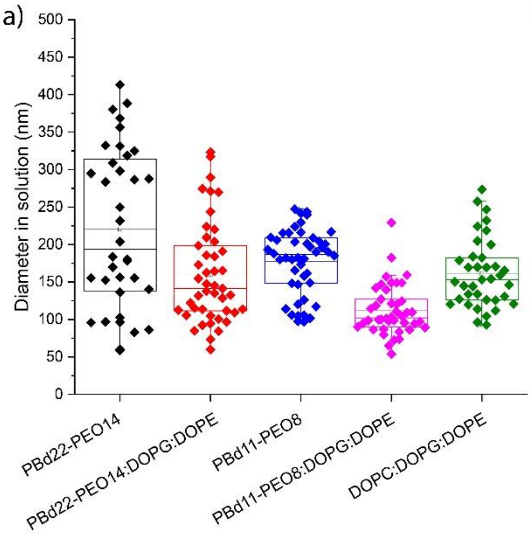

b)

Membrane composition	No.	Diameter (nm)	S.D	S.E of the mean
PBd22-PEO14	36	220.7	106.5	17.7
PBd22-PEO14:DOPG:DOPE	45	160.47	67.3	10
PBd11-PEO8	46	177.4	43.7	6.4
PBd11-PEO8:DOPG:DOPE	42	112.1	33.5	5.1
DOPC:DOPE:DOPG	37	160.9	45.7	7.5

Figure S2. Diameter of liposomes, hybrid vesicles and polymersomes as determined from AFM images.

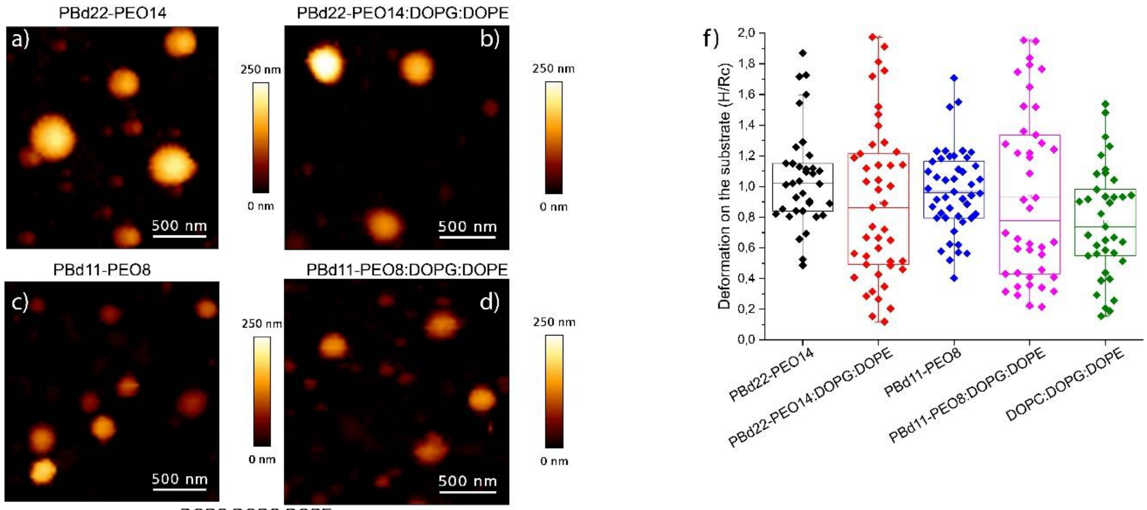

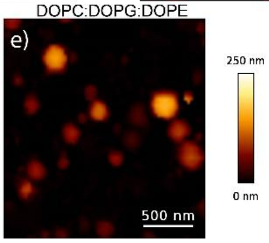

g)

Membrane composition	No	Mean (H/R_c)	SD	SE of mean	Median
PBd22-PEO14	36	1.05	0.32	0.054	1.02
PBd22-PEO14:DOPG:DOPE	45	0.89	0.50	0.08	0.86
PBd11-PEO8	46	0.97	0.27	0.04	0.96
PBd11-PEO8:DOPG:DOPE	42	0.93	0.55	0.08	0.78
DOPC:DOPE:DOPG	37	0.77	0.36	0.06	0.74

Figure S3. Characterization of the deformability of the vesicles by AFM. An overview of the topographic image using an imaging force of 80 pN of a) PBd_{22}-PEO_{14} vesicles; b) PBd_{22}-PEO_{14}:DOPG:DOPE vesicles; c) PBd_{11}-PEO_{8} vesicles; d) PBd_{11}-PEO_{8}:DOPG:DOPE vesicles; and e) DOPC:DOPG:DOPE vesicles. f–g) Deformation of the vesicles calculated as the ratio between the height (H) and the radius of curvature (Rc).

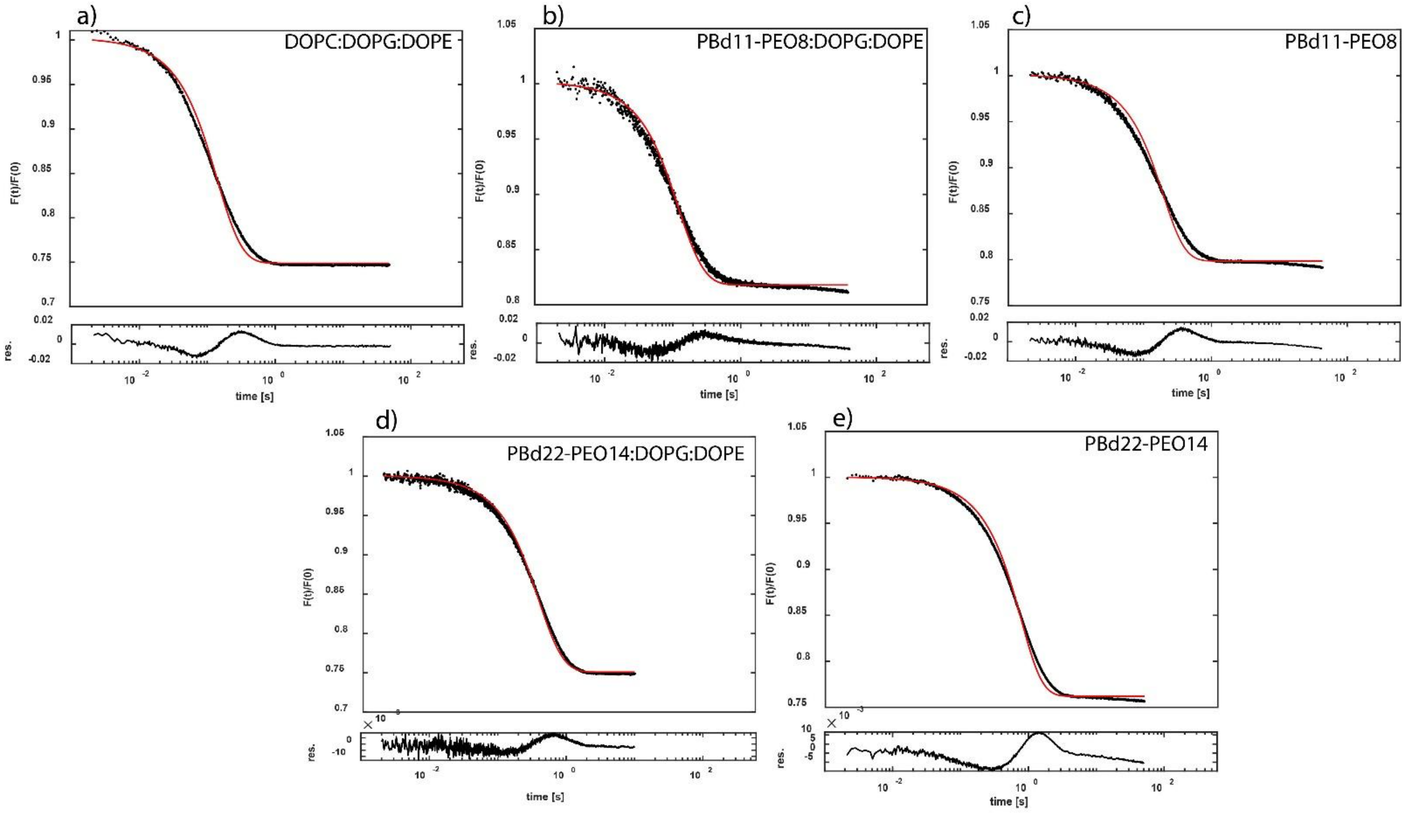

Figure S4. Fits of the calcein relaxation curves for liposomes (a), hybrid vesicles (b) and (d) and polymersomes (c) and (e). Fluorescence kinetics of vesicles osmotically shocked with 50mM KCl. The residuals are in the bottom panel. The fitting curve is in red.

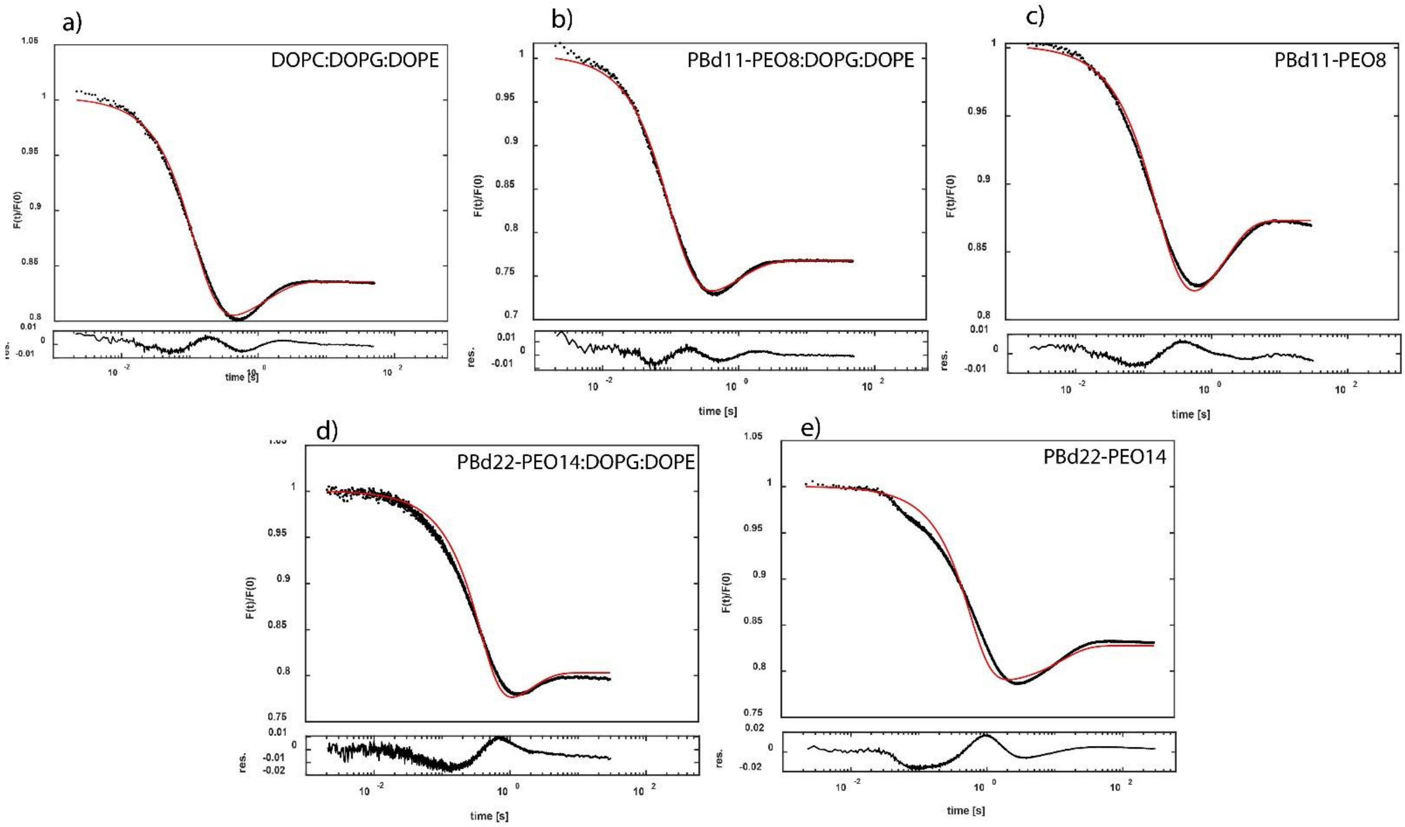

Figure Error! No text of specified style in document.**S5.** Fits of the calcein relaxation curves for liposomes (a), hybrid vesicles (b) and (d) and polymersomes (c) and (e). Fluorescence kinetics of vesicles osmotically shocked with 50mM Na-Formate. The residuals are in the bottom panel. The fitting curve is in red.

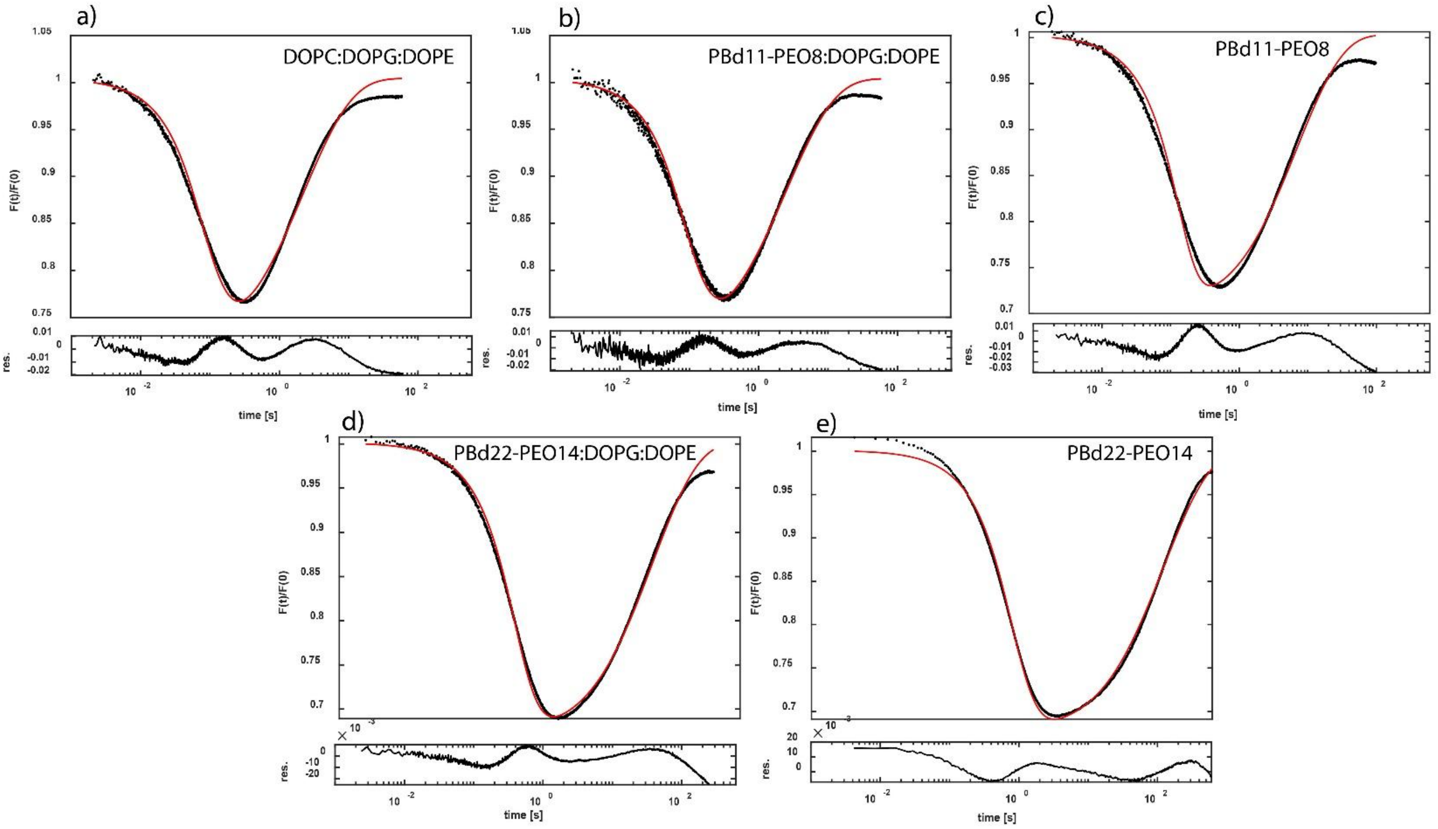

Figure S6. Fits of the calcein relaxation curves for liposomes (a), hybrid vesicles (b) and (d) and polymersomes (c) and (e). Fluorescence kinetics of vesicles osmotically shocked with 50mM Glycerol. The residuals are in the bottom panel. The fitting curve is in red.
